# The impact of framing effects, competitive state, and time pressure on risk-taking decisions in tennis players of different skill levels

**DOI:** 10.3389/fpsyg.2025.1573070

**Published:** 2025-11-04

**Authors:** Rong Shangguan, Zihan Zha

**Affiliations:** ^1^College of Physical Education, Hunan Normal University, Changsha, China; ^2^College of Education Science, Hunan Normal University, Changsha, China

**Keywords:** risk decision-making, framing effects, time pressure, competitive state, tennis players, skill levels

## Abstract

Athletes’ risk decision-making significantly influences competitive performance; however, current research remains controversial regarding how framing effects, competitive state, and time pressure affect risk decisions among athletes of different skill levels. Through two experiments, this study investigated the effects of frame type (positive/negative), competitive state (leading/trailing), and time pressure (high/low) on risk decision-making among tennis players of varying proficiency levels. Both Experiments 1 and 2 recruited 120 tennis players (40 participants each in expert, skilled, and novice groups, with a male-to-female ratio of 3:1). The findings revealed that: (1) The novice group exhibited the highest susceptibility to framing effects, maintaining this characteristic even under high time pressure; (2) the skilled group demonstrated distinctive “transitional characteristics,” showing susceptibility to framing effects without time pressure but shifting toward extremely conservative decisions under high time pressure; (3) the expert group displayed the most stable decision-making patterns, primarily basing decisions on competitive state—adopting conservative strategies when leading and aggressive strategies when trailing, with minimal influence from framing effects and time pressure. The study demonstrates significant differences in risk decision-making characteristics across skill levels: novices possess immature decision-making mechanisms and are readily influenced by emotional and external factors; skilled players are in a developmental phase of decision-making ability, exhibiting notable context dependency; whereas experts demonstrate mature decision-making mechanisms, capable of making stable strategic choices based on competitive state. These findings provide novel theoretical perspectives for understanding the developmental patterns and influencing factors of athletes’ risk decision-making.

## 1 Introduction

### 1.1 Athletes’ risk decision-making

Risk-taking decisions are a core cognitive process in which individuals weigh potential gains and losses under conditions of uncertainty. In the rapidly changing context of competitive sport, athletes must frequently make instantaneous choices that not only determine match outcomes but also shape their long-term professional development (Williams and Ward, 2007). In tennis, for instance, players must decide at critical points whether to adopt conservative or aggressive shot strategies. The quality of these decisions depends not only on technical proficiency but is also substantially shaped by psychological factors.

Skill level is a central determinant of athletes’ performance in risk-taking decisions. Compared with novices, expert athletes typically make faster and more precise choices. This advantage derives not only from extensive domain-specific knowledge (Mann et al., 2007) but also from more efficient perceptual strategies, such as optimized visual search patterns that enable rapid anticipation. By contrast, novices—owing to limited experience and training—are more susceptible to interference from external contextual factors, resulting in greater decisional uncertainty (McRobert et al., 2011). Under pressure and informational constraints, experts often rely on well-practiced, fast-and-frugal heuristics to make adaptive choices (Raab and Gigerenzer, 2015), whereas novices tend to lack such refined cognitive shortcuts.

Although research on athletic decision-making is extensive—spanning perceptual training (Abernethy et al., 2012), simple heuristics (Raab, 2012), and performance under pressure (Hepler, 2015)—the mechanisms through which these factors interact in the highly dynamic context of tennis risk-taking remain insufficiently specified. Tennis is characterized by high uncertainty and immediacy, requiring athletes to make tactical choices within split seconds (Crognier and Féry, 2005). According to the expert performance framework (Ericsson and Smith, 1991), differences in skill level should manifest in decision behavior. Yet a key question remains underexplored: how cognitive biases—exemplified by framing effects—interact with critical situational moderators such as time pressure to jointly shape the risk-taking decisions of tennis players across different skill levels.

Against this backdrop, the present study systematically examines how framing effects, competitive state, and time pressure influence the risk-taking decisions of tennis players at different skill levels. We further seek to articulate the mechanisms underlying risk-taking in tennis and to advance understanding of expert performance and decision processes in sport. Such as how to help athletes optimize their risk-taking decisions in high-pressure environments, etc.

### 1.2 The impact of framing types on athletes’ risk decision-making

The framing effect refers to systematic changes in individuals’ preferences when the same decision problem is presented in different ways (Tversky and Kahneman, 1981). In high-speed sports, the operative “frame” is often not an explicit statistic visible to athletes during competition; rather, it is implicitly constructed through contextual factors—such as coaches’ cues, athletes’ self-talk, tactical intent, local score pressure, and the immediate objective of a point or game (Wegner and Teubel, 2014). Positive framing emphasizes preserving existing gains, whereas negative framing emphasizes reducing current losses. In general, positive framing encourages lower-variance, higher-percentage choices or risk aversion, while negative framing increases risk-taking tendencies (De Martino et al., 2006).

Furthermore, skill level functions as a crucial moderating variable in the manifestation of framing effects within athletic decision-making contexts. Expert athletes, drawing upon their extensive domain-specific expertise, typically demonstrate superior capabilities in risk–benefit assessment and strategic evaluation. However, within highly competitive contexts, these athletes may paradoxically exhibit enhanced susceptibility to framing effects, primarily due to their heightened sensitivity to performance outcomes (Druckman and McDermott, 2008). Conversely, novice athletes, constrained by their limited experiential knowledge base, demonstrate reduced proficiency in risk–benefit evaluation and exhibit increased vulnerability to positive framing influences. To explain this phenomenon more deeply, this study incorporates the expertise reversal effect (Kalyuga, 2007) within the framework of Cognitive Load Theory. This theory posits that external guidance beneficial to novices, such as explicit frame cues, may constitute redundant information for experts who have already developed automated schemas, thereby increasing their extraneous cognitive load and interfering with decision-making. Thus, a significant research gap persists in understanding the mechanisms through which framing effects modulate risk decision-making processes across different skill levels, particularly in tennis.

Tennis represents a sport discipline characterized by high degrees of uncertainty and temporal immediacy, requires continuous tactical decision-making from players within rapidly evolving competitive contexts (Crognier and Féry, 2005). Current research largely focuses on team sports (such as football and basketball), while studies on tennis players’ decision-making are relatively sparse. Although many sports psychology handbooks discuss athlete decision-making, there is still a significant lack of summaries regarding decision outcomes in specific situations for tennis players. The distinctive characteristics of tennis matches, particularly regarding score-related pressure and strategic opponent interactions, suggest that frame types may significantly modulate players’ decision-making processes. Consequently, this study aims to examine the mechanisms through which frame types influence tennis players’ risk decision-making patterns, firstly, and secondly, to investigate how these effects potentially vary across different skill levels. This research will contribute to the understanding of decision-making processes in tennis.

### 1.3 The influence of frame types and competitive state on athletes’ risk decision-making processes

Competitive state, defined as the contextual positioning of an athlete’s or team’s scoring differential relative to their opponents within competitive environments (Baumeister, 1984), represents a critical factor in competitive decision-making. Empirical research demonstrates systematic variations in athletes’ risk preferences contingent upon their competitive state. Individuals in advantageous positions (leading) predominantly adopt risk-averse strategies to preserve their competitive advantage. Conversely, those in disadvantageous positions (trailing) exhibit enhanced propensity for risk-taking behaviors to minimize point differentials (Johnson et al., 2006). This systematic behavioral pattern reflects athletes’ strategic calibration of risk–benefit trade-offs across varying competitive contexts, highlighting the dynamic nature of decision-making processes in competitive sports (Gernigon et al., 2010).

Importantly, the in-match framing of options in real tennis play can shape these choices. When leading is implicitly framed as “maintaining advantage” (a positive frame), players typically eschew unnecessary risks—for example, prioritizing higher-percentage patterns such as heavy cross-court topspin, larger margins from the lines, or safer second-serve choices—to safeguard the lead. Conversely, when trailing is implicitly framed as a “must catch up” situation (a negative frame), players are more inclined to pursue higher-variance options—such as targeting closer to the lines, accelerating down-the-line winners, employing serve-plus-one aggression even behind the second serve, or initiating earlier net approaches—to narrow the deficit. Accordingly, positive framing tends to intensify conservative tendencies in leading positions, whereas negative framing amplifies risk-seeking tendencies in trailing positions (Masters et al., 2007).

While previous research has revealed the independent effects of competitive state and framing effects on athletes’ risk decision-making, their interactive influence in tennis remains unclear, particularly regarding systematic variations across different skill levels. Tennis matches are characterized by high dynamicity, requiring players to continuously adjust their decision strategies between point gains and losses. Therefore, this study aims to investigate how competitive state (leading/trailing) moderates the influence of framing effects on tennis players’ risk decision-making. Furthermore, this research will examine whether this moderating effect varies across different skill levels.

### 1.4 The effects of frame types, competitive state, and time pressure on athletes’ risk decision-making

Time pressure refers to the sense of urgency individuals experience when completing tasks under limited temporal constraints, which significantly influences both decision-making processes and outcomes (Hockey, 1997). According to Dual-Process Theory, decisions arise from the joint operation of two systems: System 1, which is fast, automatic, and heuristic-driven, and System 2, which is slower, controlled, and rule-based (Wason and Evans, 1974; Evans and Stanovich, 2013; see also Kahneman, 2011). In athletic contexts, time pressure is an inescapable situational factor—particularly in high-intensity competition—where extremely brief temporal windows compress the attentional and working-memory resources required by System 2. In time pressure situations, the influence of System 1 becomes more pronounced, requiring athletes to quickly assess risks and benefits and make decisions (Raab and Johnson, 2007). While the effective functioning of System 2 mechanisms can be difficult under such conditions, experts leverage their practical knowledge to develop heuristics and schemas, enabling them to swiftly apply their expertise and minimize decision-making errors. This is also why experts’ performance might remain largely unaffected by time pressure.

Research shows that high time pressure constrains athletes’ cognitive resources, prompting greater reliance on intuitive judgments and experiential decision-making rather than systematic analysis (Maule and Hockey, 1993). From a Dual-process perspective, this entails a shift from System-2–dominated deliberative evaluation to System-1–dominated familiar matching and rapid pattern recognition. Consequently, under high time pressure, athletes are more inclined to adopt familiar, highly accessible strategies while being more likely to overlook potentially innovative solutions (Parkin et al., 2017).

Time pressure not only directly affects athletes’ risk decisions but may also moderate framing effects across different match states. Under high time pressure, temporal constraints simplify decision processes and enhance reliance on frame-provided heuristic information, rendering athletes more sensitive to framing effects. For example, in trailing situations, high time pressure may amplify the motivational impact of negative framing on risk-taking; in leading situations, it may strengthen the influence of positive framing on conservative behavior. Conversely, under low time pressure, athletes have more time for information integration, which may attenuate framing effects and promote more rational decision-making (Ordonez and Benson, 1997).

In this study, time pressure is operationalized through experimental manipulations to simulate varying levels of temporal constraint: in the low–time pressure condition, participants’ response time is unrestricted to allow ample deliberation and engagement of System 2; in this context, the timer is not the only factor; rather, the player’s perception of being in a high-pressure situation increases the relative involvement of System 1. For example, although players may be accustomed to using a timer during their training (e.g., for serving practice), this does not necessarily trigger a high-pressure response. In fact, it is the player’s interpretation and perception of the situation that triggers the high-pressure response. Specifically, when trailing, high time pressure may further amplify the risk-promoting influence of negative framing; when leading, it may enhance the conservative influence of positive framing. Under low time pressure, longer processing time may weaken framing effects and facilitate more rational choices (Ordonez and Benson, 1997).

Moreover, time pressure may magnify the influence of competitive state on decision processes. Evidence indicates that under high time pressure, trailing competitors may adopt more extreme risk-taking strategies, whereas those who are leading tend to exhibit greater risk aversion (Hu et al., 2015). However, research on the interactive effects among time pressure, the influence of framing effects and competitive state remains limited, particularly in high-intensity sports such as tennis.

Based on previous research and theoretical analysis, this study proposes the following hypotheses: (1) Frame types, competitive state influence risk decision-making among tennis players, with effects varying across skill levels; (2) Competitive state moderates the influence of framing effects on risk decision-making, with potentially enhanced effects in trailing positions; (3) Time pressure enhances framing effects, with the sensitivity to framing varying across different competitive states and skill levels.

## 2 The impact of framing types and competitive state on tennis players’ risk decision-making

### 2.1 Methods

#### 2.1.1 Participant

Using G*Power 3.1 software (Faul et al., 2009), the required sample size for Experiment 1 was calculated based on a medium effect size (effect size *f* = 0.25), a statistical power of 0.80 (1-*β* = 0.80), and a significance level of 0.05 (*α* = 0.05). The results indicated that a minimum of 108 participants would be needed. Ultimately, 120 tennis players were recruited and divided into three groups based on their skill levels:

(1) Expert group (*n* = 40; *M* = 17.81 years, *SD* = 0.86): National Level 1 tennis players in China.
(2) Proficient group (*n* = 40; *M* = 17.58 years, *SD* = 0.13): National Level 2 tennis players in China.
(3) Novice group (*n* = 40; *M* = 19.87 years, *SD* = 0.64): Tennis students from a university in Central China who had not obtained any official certification.

All participants were right-handed, had normal or corrected-to-normal vision, and were free from color blindness, physical illnesses, or mental health disorders. Additionally, none of the participants had prior experience with similar experiments. Informed consent was obtained from each participant before the experiment, and appropriate compensation was provided afterward. This study was approved by the university ethics committee.

#### 2.1.2 Experimental design

The experiment employed a 3 (Skill Level: novice, proficient, expert) × 2 (Frame Type: positive, negative) × 2 (Competitive State: leading, trailing) mixed factorial design. Frame type and competitive state served as within-subject variables, while skill level was manipulated as a between-subject variable. The dependent variables were the percentage of risky options selected and decision-making time (Tversky and Kahneman, 1981).

#### 2.1.3 Experimental procedure

Experiment 1 was programmed and presented using E-Prime 2.0 software. The experiment was conducted in a quiet classroom free from electromagnetic interference, with all participants required to turn off their electronic devices. The experimental procedure consisted of the following phases:

(1) Preparation phase

Participants initially provided their demographic information through the E-Prime 2.0 interface. To control the influence of external pressures on experimental results, participants listened to calming music designed to facilitate relaxation.

(2) Practice phase

During the practice phase, after listening to the experimenter’s detailed procedural instructions, participants completed one practice trial. This practice session was designed to familiarize participants with the experimental procedure and response keys while reducing initial anxiety. The formal experiment commenced only after confirming that participants had fully understood the requirements and that all their questions had been addressed. Moreover, the tennis risk-decision scenario used in the practice trial differed from those in the formal experiment to ensure that it would not affect the data of the subsequent formal trials.

(3) Formal experimental phase

The experiment began with the following instruction displayed on screen: “You will encounter the following situations. There are no right or wrong choices. Please position your left index finger on the Q key and your right index finger on the P key.” Participants pressed the T key to initiate the first decision task and subsequently used the same key to proceed to each following task. Each participant completed 20 risk decision tasks, covering four combinations of 2 (frame type: positive frame, negative frame) × 2 (competition state: leading, trailing). A completion message appeared after all tasks were finished, concluding the experiment.

#### 2.1.4 Apparatus and materials

To control for extraneous variables, the experiment was conducted in a quiet classroom with consistent lighting conditions. The experimental materials were presented on a 14-inch Lenovo laptop (display resolution: 1,600 × 900 pixels) and programmed using E-Prime 2.0 software. All participants responded using the same keyboard.

The study was based on framing decision-making problems (Jordet and Hartman, 2008), which were adapted to reflect the characteristics of tennis matches (risk decision-making scenarios in tennis, including situations such as smashes, lobs, serves, returns of serve, and volleys at the net). A total of 10 formal questions and one practice question were designed. The experimental materials covered typical tennis match scenarios, including high-pressure shots, lobs, serves, returns, and net volleys. Each scenario included two decision-making options: a conservative option and a risky option. According to the framing effect theory, each scenario was presented in two formats: a positive frame (emphasizing gains) and a negative frame (emphasizing losses).

To ensure the quality of the materials, 40 sports education experts (professional tennis team coaches and university tennis instructors) from two universities were invited to evaluate the validity of the materials using a five-point Likert scale. In this scale, one indicates “very unreasonable,” two indicates “unreasonable,” three indicates “neutral,” four indicates “reasonable,” and five indicates “very reasonable.” The final results showed that 80% of the experts rated the materials as “reasonable” or “very reasonable,” indicating good content validity. The internal consistency of the materials was assessed, yielding a Cronbach’s *α* value of 0.81, which demonstrates high reliability.

To control for extraneous variables, the experiment was conducted in a quiet classroom with consistent lighting conditions. The experimental materials were presented on a 14-inch Lenovo laptop (display resolution: 1,600 × 900 pixels) and programmed using E-Prime 2.0 software. All participants responded using the same keyboard.

The study was based on framing decision-making problems (Jordet and Hartman, 2008), which were adapted to reflect the characteristics of tennis matches. A total of 10 formal questions and one practice question were designed. The experimental materials covered typical tennis match scenarios, including high-pressure shots, lobs, serves, returns, and net volleys. Each scenario included two decision-making options: a conservative option and a risky option. According to the framing effect theory, each scenario was presented in two formats: a positive frame (emphasizing gains) and a negative frame (emphasizing losses).

### 2.2 Results

#### 2.2.1 Risk-taking probability

**Figure 1:**
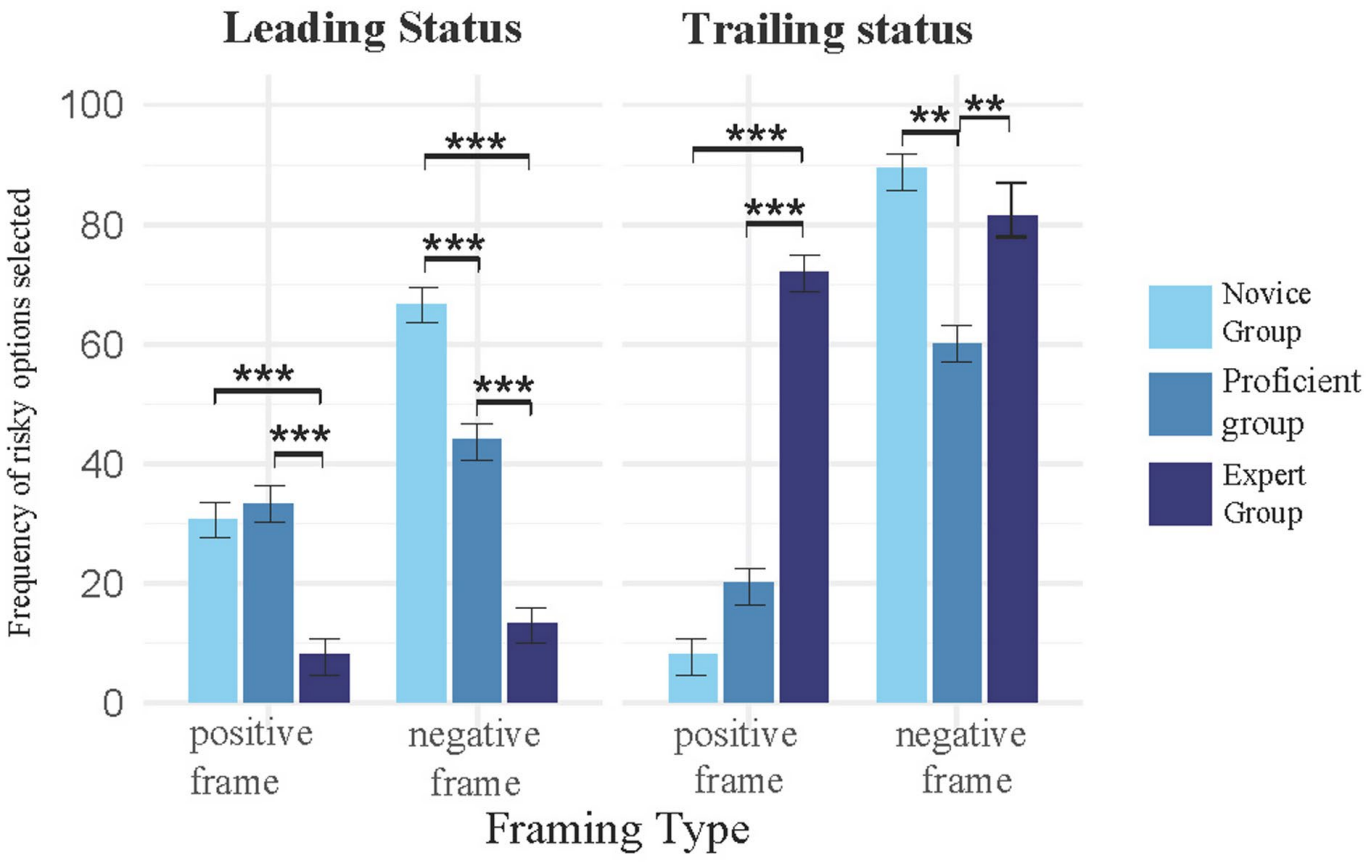
The impact of competitive state and frame type on tennis players’ risk decision-making. Error bars represent standard error of the mean (SEM). Asterisks indicate statistical significance levels: *p < 0.05, **p < 0.01, ***p < 0.001.

A 3 (Skill Level: novice/proficient/expert) × 2 (Frame Type: positive/negative) × 2 (Competitive State: leading/trailing) mixed-design analysis of variance (ANOVA) was conducted, with frame type and competitive state as within-subject variables and skill level as a between-subject variable (see Figure 1, Table 1).

**Table 1:** Descriptive statistical analysis.

	Leading status		Trailing status	
	Positive (0–100%) (*M* ± *SD*)	Negative (0–100%) (*M* ± *SD*)	Positive (0–100%) (*M* ± *SD*)	Negative (0–100%) (*M* ± *SD*)
Novice	30 ± 3.15	66 ± 3.23	7 ± 3.92	84 ± 3.34
Proficient	33 ± 3.01	43 ± 3.20	20 ± 3.56	60 ± 2.98
Expert	7 ± 3.11	13 ± 3.16	72 ± 3.38	81 ± 3.57

The results revealed a significant main effect of skill level, *F* (2, 234) = 11.95, *p* < 0.001. *Post hoc* comparisons indicated that novice athletes demonstrated significantly higher risk-taking percentages than proficient athletes (*p* < 0.001) and expert athletes (*p* = 0.022), while expert athletes exhibited slightly higher risk-taking percentages than proficient athletes, though this difference did not reach significance (*p* = 0.068). The main effect of competitive state was significant, *F* (1, 117) = 206.38, *p* < 0.001, with risk-taking percentages being significantly higher in the trailing condition compared to the leading condition. The main effect of frame type was also significant, *F* (1, 117) = 380.33, *p* < 0.001, with risk-taking percentages being significantly higher under the negative frame than the positive frame. A significant interaction effect was observed between competitive state and skill level, *F* (2, 234) = 193.93, *p* < 0.001, as well as between frame type and competitive state, *F* (1, 117) = 71.01, *p* < 0.001. Additionally, a three-way interaction effect among skill level, frame type, and competitive state was significant, *F* (2, 234) = 15.60, *p* < 0.001. Simple effects analyses demonstrated that, under the leading condition with the positive frame, no significant difference was found in risk-taking percentages between novice athletes and proficient athletes (*p* = 0.999), but novice athletes exhibited significantly lower risk-taking percentages than expert athletes (*p* < 0.001), and proficient athletes also demonstrated significantly lower risk-taking percentages than expert athletes (*p* < 0.001). Under the leading condition with the negative frame, novice athletes exhibited significantly lower risk-taking percentages than proficient (*p* < 0.001) and expert athletes (*p* < 0.001), and proficient athletes also exhibited significantly lower risk-taking percentages than expert athletes (*p* < 0.001). Under the trailing condition with the positive frame, the difference in risk-taking percentages between proficient and expert athletes approached significance (*p* = 0.0752), but novice athletes showed significantly lower risk-taking percentages than both expert (*p* < 0.001) and proficient athletes (*p* < 0.001). Under the trailing condition with the negative frame, novice athletes exhibited significantly lower risk-taking percentages than both proficient (*p* < 0.001) and expert athletes (*p* < 0.001), while no significant difference was observed between proficient and expert athletes (*p* = 0.5301).

#### 2.2.2 Decision time

**Figure 2:**
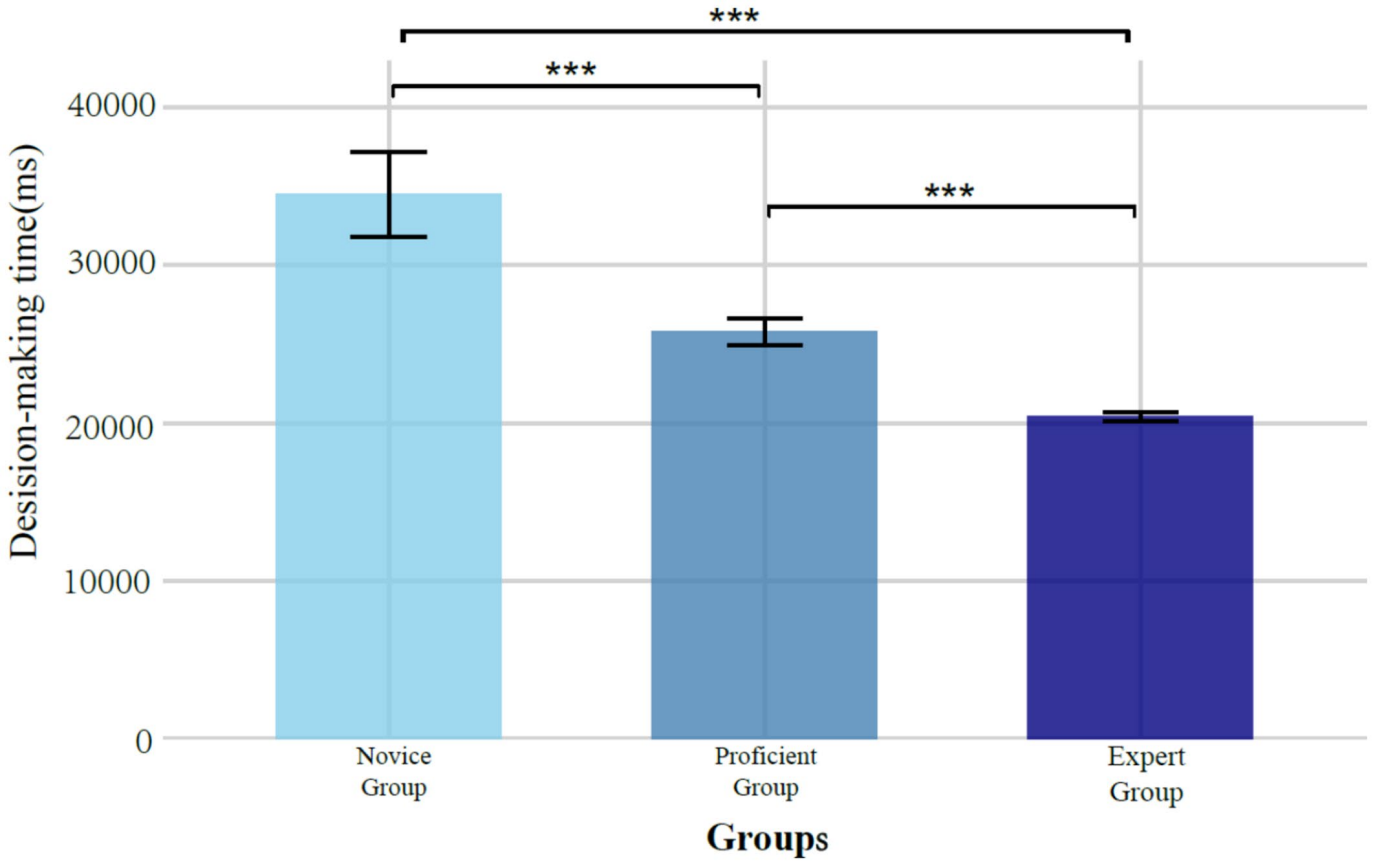
The impact of skill level on risk decision-making time in tennis players.

We conducted an analysis of variance (ANOVA) to examine the differences in decision-making time among tennis players with different skill levels. The results indicated that a significant difference among skill level groups, *F* (2, 234) = 565.73, *p* < 0.001. *Post hoc* multiple comparisons (Tukey HSD) revealed that the decision times of novice athletes were significantly longer than those of proficient athletes (*p* < 0.001) and expert athletes (*p* < 0.001). Additionally, the decision times of proficient athletes were significantly longer than those of expert athletes (*p* < 0.001). These findings suggest that decision time decreases significantly as skill level increases (see Figure 2, Table 2).

**Table 2:** The impact of skill level on risk decision-making time in tennis players.

	Novice	Proficient	Expert
Decision-making time (ms)	33,925 ± 2,548	25,867 ± 1,034	21,082 ± 514

### 2.3 Discussion

The results of Experiment 1 confirmed the significant effects of frame type and competitive state on tennis players’ risk decision-making, supporting Hypotheses 1 and 2.

The findings of Experiment 1 indicated that risk decision scores under the negative frame were significantly higher than those under the positive frame. From the perspective of Dual-Process Theory, this pattern reflects frame-contingent shifts in the dominance of two cognitive systems that guide judgment and choice (Kahneman, 2011; Evans and Stanovich, 2013). Negative frames that highlight “errors,” “losing ground,” or “falling behind” increase affective arousal and perceived urgency, thereby privileging fast, intuitive, and heuristic-driven System 1 processing. In competitive tennis, where decisions must be made within milliseconds and without access to real-time statistics, System 1 promotes actionable, expedient responses aimed at escaping aversive states—often manifesting as risk-seeking choices such as going for a lower-percentage winner or an aggressive serve to neutralize a perceived threat (Raab, 2012; Hepler and Feltz, 2012). By contrast, positive frames that emphasize “winning,” “consolidating a lead,” or “gaining an advantage” reduce threat salience and create a less urgent cognitive milieu that affords greater engagement of slow, deliberative, and resource-dependent System 2 processing. Under stronger System 2 involvement, athletes are more likely to evaluate contingencies, safeguard current advantages, and select conservative, higher-percentage options to maintain the status quo. Accordingly, the observed higher risk under negative framing can be interpreted as a frame-induced shift toward System 1 dominance, whereas the lower risk under positive framing reflects greater System 2 engagement. This Dual-process account complements Prospect Theory by specifying the cognitive–affective mechanisms through which loss-focused descriptions increase risk preference, while gain-focused descriptions promote caution in real-time sports decision-making (Tversky and Kahneman, 1981; Kahneman, 2011; Evans and Stanovich, 2013).

Under the positive frame, positive descriptions (e.g., “winning”) elicit positive emotional experiences, leading athletes to adopt more conservative strategies to maintain the status quo. Conversely, under the negative frame, negative descriptions (e.g., “errors”) trigger negative emotional experiences, motivating athletes to choose riskier strategies to escape unfavorable situations.

From the perspective of Cognitive Appraisal Theory, the negative frame influences individuals’ cognitive appraisal of the situation, thereby affecting their decision-making behavior. When athletes are exposed to a negative frame, they may evaluate the situation as a threat, which in turn elicits stronger risk-taking motivations to confront the challenge. Additionally, the negative frame may regulate athletes’ decision-making behavior by influencing their emotional states. For instance, in high-pressure situations, athletes are more susceptible to the effects of the negative frame, leading to riskier decisions (Beckmann and Goode, 2014).

Experiment 1 also revealed that risk decision scores were significantly higher in the trailing condition compared to the leading condition. In addition to the Dual-Process Theory, This finding may can be explained through several theoretical frameworks. First, according to Economic Prospect Theory, individuals exhibit risk-seeking tendencies in the domain of losses (i.e., trailing condition). When athletes are in a trailing position, they tend to perceive their current state within a “loss frame” and are more willing to adopt risky strategies to overcome their disadvantageous position in order to avoid ultimate defeat (Plessner et al., 2009).

Second, from the perspective of Regulatory Focus Theory (Higgins et al., 1997) argues that individuals are driven by two motivational orientations: promotion focus and prevention focus. When pursuing scores, athletes activate a promotion focus, emphasizing the attainment of positive outcomes. According to this theory, promotion focus concentrates on pursuing positive outcomes and visions, thereby guiding individuals to seek growth and achievement, and leading to a greater tendency to adopt aggressive risk-taking strategies. In contrast, prevention focus centers on avoiding losses and ensuring safety, resulting in more cautious and risk-averse behaviors. Therefore, athletes’ risk-taking behaviors during score pursuit exemplify the influence of promotion focus. The Regulatory Focus Theory highlights how these two different foci influence decision-making and behavior across various contexts, including achievement and motivation (Memmert et al., 2013). Furthermore, Competitive Pressure Theory suggests that trailing positions increase psychological pressure on athletes, compelling them to adopt more aggressive strategies (Jones, 1995). This pressure may trigger emotional responses such as anxiety (Lazarus, 2000), subsequently influencing decision-making behavior. For instance, research has shown that elevated anxiety levels in trailing positions cause athletes to focus more on potential gains while overlooking risks (De Heus et al., 2010).

Additionally, Experiment 1 demonstrated that novice athletes exhibited significantly longer decision times compared to both proficient and expert athletes, with proficient athletes showing significantly longer decision times than expert athletes.

## 3 Experiment 2: the effects of frame type, competitive state, and time pressure on tennis players’ risk decision-making

### 3.1 Methods

#### 3.1.1 Participants

Based on calculations using G*Power 3.1 software (Faul et al., 2009), a minimum of 108 participants was required to achieve a medium effect size (effect size *f* = 0.25), a statistical power of 0.80 (1-*β*), and a significance level of 0.05 (*α*). Ultimately, this study recruited a new cohort of 120 tennis players (none of whom had participated in Experiment 1), who were then divided into three groups based on their skill level.

(1) Expert group (*n* = 40; 30 males, 10 females; *M* = 17.23 years, *SD* = 0.72): National Level 1 tennis players in China.
(2) Proficient group (*n* = 40; 30 males, 10 females; *M* = 18.12 years, *SD* = 0.56): National Level 2 tennis players in China.
(3) Novice group (*n* = 40; 30 males, 10 females; *M* = 18.83 years, *SD* = 0.57): tennis students from a university in Central China who had not obtained any official certification.

All participants were right-handed, had normal or corrected-to-normal vision, and were free of color blindness, physical illnesses, or a history of mental disorders. None of the participants had prior experience with similar experiments. Informed consent was obtained before the experiment, and participants received an exquisite small gift afterward. This study was approved by the university ethics committee.

#### 3.1.2 Experimental design

The experiment employed a 2 (Time Pressure: no time pressure, high time pressure) × 2 (Frame Type: positive frame, negative frame) × 2 (Competitive State: leading, trailing) × 3 (Skill Level: novice, proficient, expert) four-factor mixed design. Skill Level and Time Pressure were between-subject factors, while frame type and competitive state were within-subject factors. The dependent variable was the probability of participants choosing the risky option.

#### 3.1.3 Experimental procedure

Experiment 2 was programmed using E-prime 2.0 software for stimulus presentation. While the basic procedure was similar to Experiment 1, it incorporated an additional time pressure manipulation. The specific steps were as follows:

(1) Group assignment

The 40 participants from each skill level group (novice, proficient, and expert athletes) were randomly and equally assigned to either the low or high time pressure conditions, with 20 participants per condition.

(2) Preparation phase

Identical to Experiment 1.

(3) Practice phase

Identical to Experiment 1, with the additional requirement that participants in the high time pressure condition familiarize themselves with the countdown timer constraints.

(4) Main experiment

Different instructions were presented to the low and high time pressure conditions:

Low time pressure condition

“You will encounter the following situations with no time limit for your responses. You have ample time to consider and make your choices. There are no right or wrong answers; please select what you consider to be the best option. Once you understand these instructions, please sit comfortably with your hands on the keyboard. Position your left index finger on the Q key and your right index finger on the P key.”

High time pressure condition

“You will encounter the following situations with limited response time. You must complete each task within the allocated time frame. Each item displays a countdown timer. There are no right or wrong answers; please select what you consider to be the best option. Once you understand these instructions, please sit comfortably with your hands on the keyboard. Position your left index finger on the Q key and your right index finger on the P key.”

Time constraints for the high time pressure condition were established based on the mean decision times from Experiment 1: novice athletes (31,775 ms), proficient athletes (24,934 ms), and expert athletes (20,149 ms). Tasks automatically advanced upon countdown completion. Participants were required to complete 20 risk decision-making tasks covering all four combinations of the 2 (frame type: positive/negative) × 2 (competitive state: leading/trailing) experimental conditions, following which those in the high time–pressure condition additionally completed a time–pressure manipulation check questionnaire.

#### 3.1.4 Apparatus and materials

Identical to those used in Experiment 1.

### 3.2 Results

#### 3.2.1 Time pressure manipulation check

This experiment combined a dilemma scenario in tennis, and based on previous research, used Benson and Beach’s (1996) method of determining time pressure value by recording participants’ decision-making time under no time pressure conditions, calculating the mean and standard deviation, and then reducing these values to determine the time pressure value. To verify the effectiveness of the time pressure manipulation, participants’ subjective experience of time pressure was measured using a seven-point scale (1 = “no time pressure at all,” 7 = “extreme time pressure”). An independent samples *t*-test revealed that participants in the high time pressure condition (*M* = 6.82, *SD* = 0.39) reported significantly higher levels of perceived time pressure than those in the low time pressure condition (*M* = 1.21, *SD* = 0.32), *t* (118) = 131.47, *p* < 0.001, Cohen’s *d* = 24.71. These results confirmed the effectiveness of the time pressure manipulation in this study.

#### 3.2.2 Risk-taking probability

A 3 (Skill Level: novice athletes/proficient athletes/expert athletes) × 2 (Time Pressure: high/low) × 2 (Frame Type: positive/negative) × 2 (Competitive State: leading/trailing) mixed-design analysis of variance (ANOVA) was conducted, with frame type and competitive state as within-subject factors, and skill level and time pressure as between-subject factors. The dependent variable was the probability of participants choosing the risky option.

For the main effects, the main effect of skill level was significant, *F* (2, 234) = 98.99, *p* < 0.001. *Post hoc* Tukey comparisons revealed that the risk-taking probability of proficient athletes was significantly lower than that of novice athletes (*p* < 0.001), and the probability for expert athletes was significantly higher than that of proficient athletes (*p* < 0.001). However, there was no significant difference between novice and expert athletes (*p* = 0.324). The main effect of time pressure was significant, *F* (1, 117) = 25.02, *p* < 0.001, with the risk-taking probability under high time pressure being significantly lower than under low time pressure (*p* < 0.001). The main effect of competitive state was significant, *F* (1, 117) = 346.87, *p* < 0.001, with the risk-taking probability in the trailing condition being significantly higher than in the leading condition (*p* < 0.001). The main effect of frame type was also significant, *F* (1, 117) = 100.08, *p* < 0.001, with the risk-taking probability under the negative frame being significantly higher than under the positive frame (*p* < 0.001).

**Figure 3:**
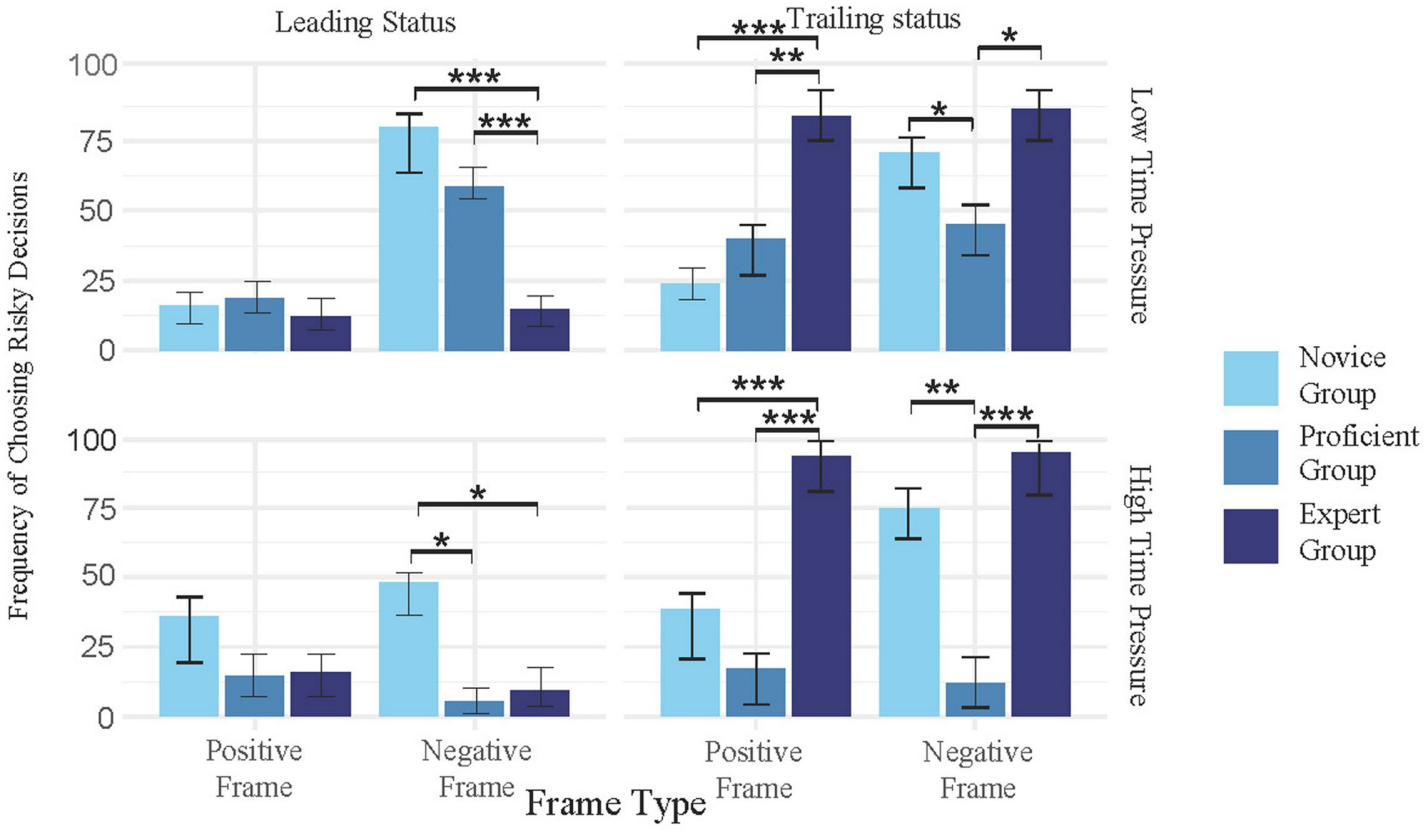
Effects of time pressure, competitive state, frame type and skill level on risk decision-making in tennis players. Error bars represent standard error of the mean (SEM). Asterisks indicate statistical significance levels: *p < 0.05, **p < 0.01, ***p < 0.001.

For interaction effects, the interaction between skill level and time pressure was significant, *F* (2, 234) = 43.75, *p* < 0.001. The interaction between skill level and competitive state was also significant, *F* (2, 234) = 231.06, *p* < 0.001. Furthermore, the interaction between time pressure and competitive state was significant, *F* (1, 117) = 6.52, *p* = 0.011, and the interaction between skill level and frame type was significant, *F* (2, 234) = 61.17, *p* < 0.001. The interaction between time pressure and frame type was also significant, *F* (1, 117) = 50.84, *p* < 0.001. However, the interaction between competitive state and frame type was not significant, *F* (1, 117) = 0.56, *p* = 0.457. For three-way interactions, the interaction among skill level, time pressure, and frame type was significant, *F* (2, 234) = 7.24, *p* < 0.001. The interaction among skill level, competitive state, and frame type was also significant, *F* (2, 234) = 4.10, *p* = 0.017. Additionally, the interaction among time pressure, competitive state, and frame type was significant, *F* (1, 117) = 21.93, *p* < 0.001. However, the interaction among skill level, time pressure, and competitive state was not significant, *F* (2, 234) = 1.56, *p* = 0.212 (Tables 3–5, Figure 3).

**Table 3:** Effects of competitive state, frame type, and skill level on risk decision-making in low time pressure in tennis players.

Type and skill level	Leading status		Trailing status	
	Positive (0–100%) (*M* ± *SD*)	Negative (0–100%) (*M* ± *SD*)	Positive (0–100%) (*M* ± *SD*)	Negative (0–100%) (*M* ± *SD*)
Novice	18 ± 4.09	80 ± 10.56	24 ± 6.05	70 ± 9.74
Proficient	19 ± 4.44	59 ± 6.87	40 ± 4.60	45 ± 5.29
Expert	12 ± 5.45	14 ± 5.88	84 ± 7.56	88 ± 6.07

**Table 4:** Effects of competitive state, frame type, and skill level on risk decision-making in high time pressure in tennis players (including means and standard deviations).

Type and skill level	Leading status		Trailing status	
	Positive (0–100%) (*M* ± *SD*)	Negative (0–100%) (*M* ± *SD*)	Positive (0–100%) (*M* ± *SD*)	Negative (0–100%) (*M* ± *SD*)
Novice	36 ± 4.02	48 ± 3.91	38 ± 8.53	75 ± 10.37
Proficient	13 ± 6.07	4 ± 3.07	18 ± 10.09	12 ± 8.06
Expert	16 ± 6.88	9 ± 5.98	92 ± 10.75	93 ± 9.67

**Table 5:** Effects of time pressure type, competitive state, frame type, and skill level on risk decision-making in tennis players.

Time pressure type	Type and skill level	Leading status		Trailing status	
		Positive (0–100%) (*M* ± *SD*)	Negative (0–100%) (*M* ± *SD*)	Positive (0–100%) (*M* ± *SD*)	Negative (0–100%) (*M* ± *SD*)
Low time pressure	Novice	18 ± 4.09	80 ± 10.56	24 ± 6.05	70 ± 9.74
	Proficient	19 ± 4.44	59 ± 6.87	40 ± 4.60	45 ± 5.29
	Expert	12 ± 5.45	14 ± 5.88	84 ± 7.56	88 ± 6.07
High time pressure	Novice	36 ± 4.02	48 ± 3.91	38 ± 8.53	75 ± 10.37
	Proficient	13 ± 6.07	4 ± 3.07	18 ± 10.09	12 ± 8.06
	Expert	16 ± 6.88	9 ± 5.98	92 ± 10.75	93 ± 9.67

For four-way interactions, the interaction among skill level, time pressure, competitive state, and frame type was significant, *F* (2, 234) = 3.18, *p* = 0.043. Under the low time–pressure condition, in the leading condition with the negative frame, the risk-taking probability of novice athletes was significantly higher than that of proficient athletes (*p* < 0.001) and expert athletes (*p* < 0.001), and proficient athletes exhibited significantly higher risk-taking probabilities than expert athletes (*p* < 0.001). In the positive frame, no significant differences were observed among the three groups (novice vs. proficient: *p* = 0.581; novice vs. expert: *p* = 0.461; proficient vs. expert: *p* = 0.198). In the trailing condition, under the positive frame, the risk-taking probability of expert athletes was significantly higher than that of novice athletes (*p* < 0.001) and proficient athletes (*p* < 0.001), and novice athletes exhibited significantly lower probabilities than proficient athletes (*p* = 0.003).

Under the negative frame, expert athletes demonstrated significantly higher risk-taking probabilities than novice athletes (*p* = 0.003) and proficient athletes (*p* < 0.001), while novice athletes exhibited significantly higher probabilities than proficient athletes (*p* < 0.001).

Under the high time–pressure condition, in the leading condition with the negative frame, novice athletes had significantly higher risk-taking probabilities than proficient athletes (*p* < 0.001) and expert athletes (*p* < 0.001), while no significant difference was found between proficient and expert athletes (*p* = 0.461). In the positive frame, novice athletes still exhibited significantly higher probabilities than proficient athletes (*p* < 0.001) and expert athletes (*p* < 0.001), but no significant difference was observed between proficient and expert athletes (*p* = 0.854). In the trailing condition, under the positive frame, expert athletes demonstrated significantly higher risk-taking probabilities than novice athletes (*p* < 0.001) and proficient athletes (*p* < 0.001), and novice athletes exhibited significantly higher probabilities than proficient athletes (*p* < 0.001). Under the negative frame, expert athletes also had significantly higher risk-taking probabilities than novice athletes (*p* < 0.001) and proficient athletes (*p* < 0.001), and novice athletes exhibited significantly higher probabilities than proficient athletes (*p* < 0.001).

### 3.3 Discussion

The experimental results demonstrated that time pressure significantly influences tennis players’ risk decision-making, with this effect varying according to athletes’ skill levels. Generally, under high time pressure conditions, athletes exhibited more conservative decision-making tendencies. This finding can be explained from both cognitive processing and stress coping perspectives.

From a cognitive processing perspective, according to Cognitive Load Theory (Sweller et al., 2019), time pressure consumes athletes’ cognitive resources, leading them to favor safer and more familiar strategies. This is particularly evident among proficient athletes who, under high time pressure, tend to adopt well-mastered conservative playing styles rather than attempting risky strategies with uncertain outcomes. This finding aligns with Zur and Breznitz’s (1981) research, which found that decision-makers tend to simplify their decision-making processes and choose more secure options under time pressure.

From a stress coping perspective, time pressure induces anxiety in athletes (Lazarus and Folkman, 1984). According to Cognitive Appraisal Theory, when individuals appraise time pressure as a threat, they exhibit defensive responses manifesting as more cautious and conservative decision-making tendencies. This is particularly notable among proficient athletes who, under high time pressure, become more focused on avoiding negative outcomes such as “errors” and “losing the match.”

However, the impact of time pressure manifests differently across athletes of varying expertise. Under high time pressure, novice athletes experience substantial cognitive challenges, simultaneously managing technical execution and contextual cues. As working-memory resources are constrained, novices become more susceptible to framing effects and tend to make intuitive, higher-variance choices. In contrast, expert athletes leverage well-developed cognitive schemas that enable more efficient information processing and decision-making under temporal constraints (Ericsson and Smith, 1991).

## 4 General discussion

Through two experiments, this study examined how framing type, time pressure, and competitive state affect risk decision-making preferences among tennis players with different skill levels. The results indicate that both framing effects and time pressure significantly influence tennis players’ risk decisions, and athletes at different skill levels display markedly different decision preferences.

### 4.1 The impact of framing effects on tennis players’ risk decision-making

Experiment 1 verified the significant impact of framing effects on tennis players’ risk decisions. Under conditions without time pressure, both novice and proficient athletes exhibited typical framing effects: conservative choices under positive frames and risk-seeking choices under negative frames. This finding aligns with Tversky and Kahneman’s (1981) classic framing effect theory, which proposes that individuals tend to be risk-averse under positive frames and risk-seeking under negative frames. This phenomenon may be attributed to distinct emotional experiences elicited by different frame types. In positive frames, descriptions such as “winning the match” evoke positive emotions, leading athletes to maintain the status quo to preserve current satisfaction. Positively framed information activates the brain’s reward system, creating a sense of satisfaction that athletes seek to protect through risk-averse choices. This is consistent with Cognitive Appraisal Theory, which suggests that positive emotional states prompt individuals to evaluate potential losses more negatively than equivalent gains, resulting in more conservative decisions to maintain their positive state. Conversely, in negative frames, descriptions of “errors” or “losing the match” trigger negative emotions, prompting athletes to adopt riskier strategies to overcome unfavorable situations and alleviate negative emotional states.

However, expert athletes’ risk decisions were not significantly influenced by framing effects, a finding that can be compellingly explained by Dual-Process Theory (Evans, 2008; Evans and Stanovich, 2013). Under time pressure, the mechanisms of System 2 often struggle to function, while efficient decision-making requires the involvement of these mechanisms. Extensive professional training seems to foster highly automated decision-making processes, with experts primarily relying on a fast, intuitive “System 1” mode of thinking. Due to their long-term practice, experts develop heuristics that encapsulate their knowledge. This system operates through heuristics derived from extensive competitive experience, enabling experts to rapidly recognize key patterns and optimal responses, thereby utilizing their knowledge effectively. Consequently, it is less sensitive to emotional fluctuations induced by surface-level linguistic frames (e.g., “winning” vs. “errors”), which typically engage and bias System 2. In contrast, the decisions of novice and proficient athletes may depend more on the slower, analytical “System 2,” which is more vulnerable to cognitive and emotional biases introduced by framing.

### 4.2 Interactive effects of framing and competitive state on tennis players’ risk decision-making

In both Experiment 1 and Experiment 2, competitive state and framing significantly influenced tennis players’ risk decisions, revealing complex interactions between competitive state, framing, and skill level that challenge previous theoretical assumptions.

The experimental results can be compellingly explained by Dual-Process Theory (Evans, 2008, 2009; Evans and Stanovich, 2013) and the Expertise Reversal Effect (Kalyuga, 2007). According to Dual-Process Theory, decision-making behavior varies substantially across different skill levels, with experts demonstrating a more sophisticated use of the intuitive system (System 1) compared to novices.

Particularly intriguing was the differential response to framing across competitive states. In leading scenarios, novices showed unexpected variability in risk-taking behaviors, with marked differences between positive and negative frames. This suggests that novices’ decision-making is more context-sensitive and emotionally influenced than previously theorized.

The Expertise Reversal Effect becomes particularly evident when examining the responses across skill levels. In high-complexity contexts, such as trailing states, the cognitive processing of athletes differs dramatically. While novices demonstrated significant fluctuations in risk-taking strategies, experts maintained a more consistent approach across different framing conditions.

High-pressure scenarios further illuminated the cognitive differences between skill levels. Experts appeared to transcend surface-level framing effects, relying instead on deeply ingrained, experience-driven decision-making strategies. In contrast, novices showed greater susceptibility to contextual cues and emotional framing.

The findings challenge the simplistic assumption of linear risk-taking behaviors. Instead, they reveal a nuanced picture of how athletes at different skill levels process competitive information. Novices’ decision-making appears to be more dynamic and context-dependent, with their risk-taking strategies significantly influenced by both competitive state and framing.

Importantly, the results suggest that the transition from novice to expert involves more than merely acquiring technical skills. It represents a fundamental transformation in cognitive processing, enabling athletes to develop more sophisticated and flexible strategic thinking across various competitive scenarios.

The study contributes to our understanding of decision-making under pressure by demonstrating how expertise fundamentally alters cognitive approaches. Experience does not simply improve performance; it transforms the very way athletes perceive and respond to competitive challenges.

### 4.3 The impact of time pressure on tennis players’ risk decision-making

Experiment 2 further examined risk decision-making under time pressure among athletes of different skill levels. The results showed that, under high time pressure, tennis players generally became more conservative. This finding strongly supports Cognitive Load Theory (Sweller et al., 2019): temporal constraints increase intrinsic and extraneous load, compress working-memory capacity, and push athletes toward familiar, lower-risk strategies. Time pressure also elevates arousal/anxiety; when appraised as threatening, athletes display defensive responses characterized by caution, further reinforcing the conservative shift.

Skill-level differences under time pressure are consistent with the Expertise Reversal Effect (Kalyuga, 2007; Kalyuga et al., 2003). Novices must handle technical execution and contextual interpretation simultaneously; under high load, their underdeveloped System 1 struggles to stabilize decisions, while the resource-intensive System 2 is easily disrupted, making them more susceptible to framing and performance instability. Proficient athletes are in a transition from analytical control to automated control; time pressure sharply reduces analytical resources while intuitive schemas are not yet robust, leading them to revert to the “simplest and safest” solutions to avoid errors. By contrast, experts possess highly automated schemas and stable intuitive routines that run with minimal additional cognitive burden, allowing them to make consistent, context-aligned decisions even under strict time constraints and to be relatively insensitive to surface-level framing (Raab and Johnson, 2007; Williams and Ward, 2007). Therefore, as expertise increases, the “locus” of limitation shifts from working-memory bottlenecks to strategic adaptation aligned with task goals.

### 4.4 Limitations and future directions

While this study provides valuable insights into the influence of time pressure on risk-taking decisions among athletes of varying expertise, several limitations must be acknowledged. The primary limitation concerns the ecological validity of the study. By simulating pressure through reaction time constraints, we captured a key element of competitive sports (Raab and Johnson, 2007); however, this approach fails to fully replicate the complex subjective pressure experienced in real-world competitions. Authentic competitive pressure is a multifaceted construct arising from a confluence of cognitive load, emotional arousal, and social-evaluative factors, which our singular manipulation did not fully encompass. During the experimental preparation phase, although participants listened to relaxing music to mitigate extraneous environmental influences, no manipulation check was conducted to formally quantify their baseline state of relaxation. Additionally, regarding the experimental procedure, the description of the “expertise” of the sports education experts who participated in the assessment of the scenarios should be included in the procedure section.

To address these shortcomings, we propose two primary directions for future research. First, it is imperative to enhance the ecological validity of the pressure manipulation, shifting the focus from an isolated “time pressure” paradigm to inducing a more holistic “contextualized subjective pressure.” Future experiments could adopt a multi-sensory, context-rich paradigm, such as presenting video clips of critical game moments, supplemented with simulated crowd noise and real-time score feedback. This would more effectively elicit the complex psychological state experienced by athletes in authentic competitive settings, thereby strengthening the generalizability of the findings. Second, future studies must increase procedural rigor by incorporating baseline measurements and manipulation checks. To accurately assess the efficacy of the pressure manipulation and control for individual differences, researchers should employ a combination of standardized subjective scales (e.g., STAI, VAS) and objective physiological indicators (e.g., Heart Rate Variability, HRV) to quantify participants’ baseline states prior to the experimental task.

## 5 Conclusion

This study revealed that framing effects and time pressure play crucial roles in risk decision-making among tennis players of different skill levels:

(1) Novice athletes: they demonstrated the highest susceptibility to framing effects, maintaining this characteristic even under high time pressure, reflecting immature decision-making mechanisms that are easily influenced by emotional and external factors.
(2) Proficient athletes: they exhibited unique “transitional characteristics,” being influenced by framing effects under no time pressure but shifting toward conservative decisions under high time pressure, potentially reflecting their developing decision-making capabilities.
(3) Expert athletes: they demonstrated the most stable decision-making patterns, making decisions primarily based on competitive state rather than framing effects or time pressure, reflecting mature decision-making mechanisms.

Novice athletes were susceptible to both framing effects and time pressure; proficient athletes were more significantly influenced by time pressure, with their decision-making patterns varying according to pressure levels; expert athletes effectively resisted the interference of both framing effects and time pressure, maintaining stable decision-making patterns. However, expert athletes’ risk decisions were primarily influenced by competitive state rather than framing effects or time pressure, specifically manifesting as conservative strategies when leading and aggressive strategies when trailing.

## Data Availability

The datasets presented in this study can be found in online repositories. The names of the repository/repositories and accession number(s) can be found in the article/Supplementary material.
