# Supplementary material for: The impact of framing effects, competitive state, and time pressure on risk-taking decisions in tennis players of different skill levels
Source: Front Psychol. 2025 Nov 4;16:1573070. doi: 10.3389/fpsyg.2025.1573070 (PMC12624778; doi:10.3389/fpsyg.2025.1573070)
Supplement: Supplementary file 1 [file Data_Sheet_1.pdf]

Appendix 1: Risk-Taking and Decision-Making Scenarios in Tennis

Scenario 1

Situation: You are in a best-of-three sets singles match. The score is 40-40 (deuce), and you are leading in games, 2-0 or 2-1. Your opponent has just hit a lob to you. Which of the following two options do you choose?

Positive Framing:

(P) Hitting a lob back has a 90% chance of success, with a 50% chance of winning the point.

(Q) Hitting an overhead has a 50% chance of success, with a 90% chance of winning the point.

Negative Framing:

(P) By keeping your error rate on the lob to around 10%, your opponent has a 50% chance to score.

(Q) By keeping your error rate on the overhead to around 50%, your opponent has a 10% chance to score.

Scenario 2

Situation: You are in a best-of-three sets singles match. The score is 40-40 (deuce), and you are trailing in games, 2-0 or 2-1. Your opponent has just hit a lob to you. Which of the following two options do you choose?

Positive Framing:

(P) Hitting a lob back has a 90% chance of success, with a 50% chance of winning the point.

(Q) Hitting an overhead has a 50% chance of success, with a 90% chance of winning the point.

Negative Framing:

(P) By keeping your error rate on the lob to around 10%, your opponent has a 50% chance of winning the point.

(Q) By keeping your error rate on the overhead to around 50%, your opponent has a 10% chance of winning the point.

Scenario 3

Situation: You are in a best-of-three sets singles match. The score is 40-40 (deuce), and you are leading in games, 2-0 or 2-1. It is your turn to serve. Which of the following two options do you choose?

Positive Framing:

(P) A second serve has a 90% chance of going in, with a 50% chance of winning the point.

(Q) A first serve has a 50% chance of going in, with a 90% chance of winning the point.

Negative Framing:

(P) By keeping your second serve error rate to around 10%, your opponent has a 50% chance of making an effective attack.

(Q) By keeping your first serve error rate to around 50%, your opponent has a 10% chance of making an effective attack.

Scenario 4

Situation: You are in a best-of-three sets singles match. The score is 40-40 (deuce), and you are trailing in games, 2-0 or 2-1. It is your turn to serve. Which of the following two options do you choose?

Positive Framing:

(P) A second serve has a 90% chance of going in, with a 50% chance of winning the point.

(Q) A first serve has a 50% chance of going in, with a 90% chance of winning the point.

Negative Framing:

(P) By keeping your second serve error rate to around 10%, your opponent has a 50% chance of winning the point.

(Q) By keeping your first serve error rate to around 50%, your opponent has a 10% chance of winning the point.

Scenario 5

Situation: You are in a best-of-three sets singles match. The score is 40-40 (deuce), and you are leading in games, 2-0 or 2-1. Your opponent's return lands near the service line. What do you choose?

Positive Framing:

(P) Hitting a deep return to the baseline has a 90% chance of success, with a 50% chance of winning the point.

(Q) Coming to the net for a volley has a 50% chance of success, with a 90% chance of winning the point.

Negative Framing:

(P) By keeping your error rate on the baseline return to around 10%, your opponent has a 50% chance to score.

(Q) By keeping your error rate on the net volley to around 50%, your opponent has a 10% chance to score.

Scenario 6

Situation: You are in a best-of-three sets singles match. The score is 40-40 (deuce), and you are trailing in games, 2-0 or 2-1. Your opponent's return lands near the service line. What do you choose?

Positive Framing:

(P) Hitting a deep return to the baseline has a 90% chance of success, with a 50% chance to score.

(Q) Coming to the net for a volley has a 50% chance of success, with a 90% chance to score.

Negative Framing:

(P) By keeping your error rate on the baseline return to around 10%, there is a 50% chance you will lose the point.

(Q) By keeping your error rate on the net volley to around 50%, your opponent has a 10% chance of winning the point.

Scenario 7

Situation: You are in a best-of-three sets doubles match. The score is 40-40 (deuce), and your team is leading in games, 2-0 or 2-1. The opponent faults on their first serve and their second serve is in. What do you choose?

Positive Framing:

(P) Blocking the return to the baseline has a 90% chance of going in, with a 50% chance of winning

the point.

(Q) Hitting a drop shot to the net has a 50% chance of success, with a 90% chance of winning the point.

Negative Framing:

(P) By keeping your error rate on the block return to around 10%, your opponent has a 50% chance to score directly.

(Q) By keeping your error rate on the drop shot to around 50%, your opponent has a 10% chance to score directly.

Scenario 8

Situation: You are in a best-of-three sets doubles match. The score is 40-40 (deuce), and your team is trailing in games, 2-0 or 2-1. The opponent faults on their first serve and their second serve is in. What do you choose?

Positive Framing:

(P) Blocking the return to the baseline has a 90% chance of going in, with a 50% chance of scoring directly.

(Q) Hitting a drop shot to the net has a 50% chance of success, with a 90% chance of scoring directly.

Negative Framing:

(P) By keeping your error rate on the block return to around 10%, there is a 50% chance you will lose the point.

(Q) By keeping your error rate on the drop shot to around 50%, there is a 10% chance you will lose the point.

Scenario 9

Situation: You are in a best-of-three sets doubles match. The score is 40-40 (deuce), and your team is leading in games, 2-0 or 2-1. Your opponent is serving, having just missed consecutive serves out wide. What do you choose?

Positive Framing:

(P) Maintaining a normal return position gives you a 90% chance of a successful return, with a 50% chance of winning the point.

(Q) Shifting your position to cover the T (center line) gives you a 50% chance of a successful return, with a 10% chance of winning the point.

Negative Framing:

(P) From a normal return position, there is a 10% chance of an error, and the opponent has a 50% chance to score.

(Q) By shifting to cover the T, there is a 50% chance of an error, and the opponent has a 10% chance to score directly.

Scenario 10

Situation: You are in a best-of-three sets doubles match. The score is 40-40 (deuce), and your team is trailing in games, 2-0 or 2-1. Your opponent is serving, having just missed a first serve out wide. What

do you choose?

Positive Framing:

(P) Maintaining a normal return position gives you a 90% chance of a successful return, with a 50% chance to turn the situation around.

(Q) Shifting your position to cover the T gives you a 50% chance of making an effective attack, with a 90% chance to turn the situation around.

Negative Framing:

(P) From a normal return position, there is a 10% chance of an error, with a 50% chance of losing the point.

(Q) By shifting to cover the T, there is a 50% chance of an error, and the opponent has a 90% chance of winning the point.
